# The effect of purified hemoglobin spray on the treatment of pediatric patients with second-degree burns

**DOI:** 10.3389/fped.2026.1744372

**Published:** 2026-02-20

**Authors:** Erol Can Kulice

**Affiliations:** Department of Pediatric Surgery, Antalya Training and Research Hospital, Antalya, Türkiye

**Keywords:** epithelialization, hemoglobin spray, pediatric burn, scar prevention, wound care

## Abstract

**Introduction:**

Purified hemoglobin spray had emerged as a potential adjunctive therapy to accelerate wound healing in pediatric patients with burns. This retrospective study aimed to compare the healing process and post-treatment outcomes of children with second-degree burns treated with or without purified hemoglobin spray in a tertiary burn unit between December 1, 2023, and December 1, 2024. Data including demographics, burn characteristics (source, percentage, depth), number of dressings, length of hospital stay, and the day of complete epithelialization were recorded. Follow-up assessments at 3 and 6 months post-discharge evaluated the presence of pruritus and epithelialization defects, including hypertrophic scarring, pigmentation changes, or contractures.

**Methods:**

Data were collected from pediatric patients with second-degree burns treated in a tertiary burn unit between December 1, 2023, and December 1, 2024. Variables included demographics, burn characteristics (source, percent TBSA, depth), number of dressings, length of hospital stay, and day of complete epithelialization. Patients were grouped by receipt of 99.9% purified hemoglobin spray versus no spray. Follow-up assessments at 3 and 6 months post-discharge evaluated pruritus and epithelialization defects, including hypertrophic scarring, pigmentation changes, or contractures.

**Results:**

Patients treated with 99.9% purified hemoglobin spray had significantly shorter hospital stays and epithelialization times (*p* < 0.001). Post-discharge pruritus was also significantly less common in this group (*p* < 0.001). The risk of developing pigmentation defects and epithelialization defects at the third month after discharge was reduced by 94.0% and 90.9%, respectively, while at the sixth month the risk of pigmentation defects was reduced by 90.9% and the risk of hypertrophic scar development during the 6-month follow-up period was reduced by 93.3%.

**Discussion:**

These findings suggested that purified hemoglobin spray might enhance wound healing, reduce the need for frequent dressing changes, and minimize long-term complications in pediatric burn care.

## 1 Introduction

In burned tissue, microvascular perfusion was impaired due to the increase in inflammatory mediators released from the tissue immediately after trauma, and correction of this damage was important for the healing of the burn area (1, 2). In addition, as the levels of free radicals such as superoxide anion, hydroxyl radical, hydrogen peroxide, and peroxynitrite increased in the burned tissue, tissue oxygenation was impaired. Therefore, the healing of the burn area also depended on increasing the oxygenation of the damaged tissue and removing free radicals from the environment (3).

During the burn healing process, depending on the depth and extent of the burn area, patients with burns might have required hospitalized and treated in burn units. Burns were among the traumas that cause both physical and psychosocial effects and, in this respect, the cost of treatment was high (1). Although the length of hospital stay of patients admitted to burn units varied, studies reported that the mean duration of hospitalization ranged between 5 and 17 days (4–7). The variation in this period depended on factors such as the depth and extent of the burn, infections developing during treatment, the presence of comorbidities, and the use of appropriate burn care products.

In recent years, various burn care products were developed to accelerate epithelialization in burn treatment. Chlorhexidine-impregnated paraffin gauze, silver-containing dressings, and biosynthetic products were commonly preferred. With these developments, it was reported that inpatients recovered with an average of 3–8 dressing changes (8–10).

Closure of the burn area by epithelialization didn't mean that the treatment was complete, because 16% to 46% of treated patients might have developed epithelialization defects such as hypertrophic scars or contractures. Hypertrophic scars were seen in 30% to 90% of the general burn population, and the vast majority of these cases occured in pediatric patients with burns. The general consensus was that burn areas healed within three weeks (9–11). It was known that delayed healing played a role in the development of hypertrophic scars (12, 13). However, even within this accepted healing period, hypertrophic scars occured at a high rate. Studies indicated that the shorter the epithelialization period, the lower the risk of hypertrophic scar formation (12). It was known that fibroblast density and irregular collagen content were increased in hypertrophic scar tissue (13–15). Normal collagen synthesis in the damaged tissue in an orderly structure was possible through hydroxylation of lysine and proline. Thus, the development of hypertrophic scars in burned tissues could be prevented, and healthy wound healing could be achieved (16).

Although it was thought that the treatment was complete when epithelialization was achieved in burns, one of the most common complaints of patients after discharge was pruritus. Although it was more common in large-area burns, it was also been seen in small burns and might have persisted for up to two years. Pruritus was generally more frequent in burns that healed with epithelialization defects such as hypertrophic scars or grafted areas, but it occured in two-thirds of pediatric patients with burns. The type of pruritus seen in patients with burns was neuropathic pruritus, and its mechanisms of occurrence were peripheral sensitization and the intact nociceptor hypothesis (17). Multiple methods were used for its treatment. There were studies reporting that the severity of pruritus was independent of age, sex, burn area, and burn etiology (8, 18–23).

There were several methods to ensure proper and rapid wound healing. One of these was the topically applicable 99.9% purified hemoglobin spray. Topical 99.9% purified hemoglobin spray accelerated the wound-healing process by increasing oxygenation on the wound surface through facilitated diffusion. Purified hemoglobin spray was used in wounds with prolonged healing periods such as burns, venous ulcers, diabetic foot ulcers, infected post-traumatic, and postoperative wounds, and there were studies showing that it accelerated the healing process (4, 12, 23, 24). However, studies on the use of 99.9% purified hemoglobin spray in patients with burns were quite limited (4).

The aim of this study was to comparatively evaluate the healing processes and post-treatment follow-up outcomes of pediatric patients in our burn unit who were treated with conventional dressings alone and those with superficial second-degree burns who received 99.9% purified hemoglobin spray in addition to conventional dressings starting from the first dressing.

## 2 Methods

### 2.1 Study design and ethical approval

This retrospective, single-center case-control study was conducted to evaluate the effect of 99.9% purified hemoglobin spray in addition to standard burn dressings on wound healing outcomes. Pediatric patients under the age of 18 who were hospitalized and treated for burn injuries at the Antalya Training and Research Hospital Burn Unit between December 1, 2023, and December 1, 2024, were included. The study was approved by the local ethics committee (Approval No: 2024-429).

### 2.2 Data collection

Data were obtained from the hospital information system and included demographic information, burn etiology, burn percentage, degree and depth, type and number of dressings, surgical interventions (debridement under anesthesia, escharotomy, grafting, or flap procedures), wound culture results, length of hospital stay, and day of complete epithelialization.

### 2.3 Clinical evaluation and patient management

The total burn surface area, burn depth, and the need for surgical intervention were evaluated by a single-blinded clinician through clinical observation. In all pediatric patients with burns admitted to the burn unit, daily caloric and protein requirements were routinely calculated, and nutritional support was provided. In patients with burns involving mobile joint areas, a rehabilitation program was initiated based on the clinician's observations.

### 2.4 Purified hemoglobin spray characteristics and application

The purified hemoglobin spray used in this study was supplied to our hospital as a wound and burn spray and was routinely available for patient use. No observational or quantitative method was used to assess the local increase in tissue oxygenation attributed to the purified hemoglobin spray. The decision for discharge was made by the clinician following direct observation of the burned areas.

### 2.5 Follow-up and outcome assessment

Post-discharge follow-ups at the early period, and at the 3rd and 6th months, were performed by a single-blinded clinician through clinical observation, assessing the presence of pruritus and epithelialization defects such as hypertrophic scarring, pigmentation differences, or contractures in the healed areas affected by burns.

### 2.6 Control group (Group 1)

The control group (Group 1) was composed of patients identified through the hospital information system who had superficial second-degree burns and received standard wound care using paraffin-impregnated gauze only. After cleansing the wounds with appropriate antiseptic solutions, all burn surfaces were covered with chlorhexidine-impregnated paraffin gauze. Epithelialization status in these patients was evaluated by a single-blinded clinician through clinical observation during dressing changes performed every two days. Routine burn dressings were performed in our unit's dressing rooms, in separate rooms for each patient under hygienic conditions, without the need for anesthesia.

### 2.7 Case group (Group 2)

The case group (Group 2) consisted of patients with superficial second-degree burns who received 99.9% purified hemoglobin spray in addition to classical burn dressings starting from the first dressing. After cleansing the wound with appropriate antiseptic solutions, purified hemoglobin spray was applied to all burn surfaces, followed by coverage with chlorhexidine-impregnated paraffin gauze. The 99.9% purified hemoglobin spray was a product manufactured by Mölnlycke Health Care AB® under the trade name Granulox®. It contained 10% carbonylated hemoglobin, 0.7% phenoxyethanol, 0.9% sodium chloride, 0.05% N-acetylcysteine, and water. As written in the product leaflet and in the references used in our study, the product was stored under cold-chain conditions before the first use and was applied at each dressing change, which was done every 3 days. After the first use, no special storage conditions were needed. The spray was applied as a 1-second puff from a distance of 10 cm to a wound area of 2.5 cm × 2.5 cm. This use was about 0.4 mL per application. The same product was used for each patient during the whole treatment period. However, if the first patient was discharged and the product was still within its shelf life, it continued to be used, because it was stored properly and did not touch the patient directly, so sterility was not affected.

### 2.8 Discontinuation and safety assessment

Once epithelialization was observed based on clinical assessment, the application of purified hemoglobin spray was discontinued.

### 2.9 Patients selection and study population

To ensure homogeneity, both groups were matched based on burn etiology, degree, percentage, depth, nutritional support, rehabilitation support, and presence of comorbidities. A total of 77 patients were treated during the study period; however, 11 patients who did not attend follow-up visits and 7 patients with incomplete data were excluded.

### 2.10 Statistical analysis

For statistical analysis, the Mann–Whitney U test, chi-square test, logistic regression, and generalized linear models (GLM) were used. The significance level was set at *p* < 0.05.

## 3 Results

### 3.1 Study population and baseline characteristics

**Figure 1:**
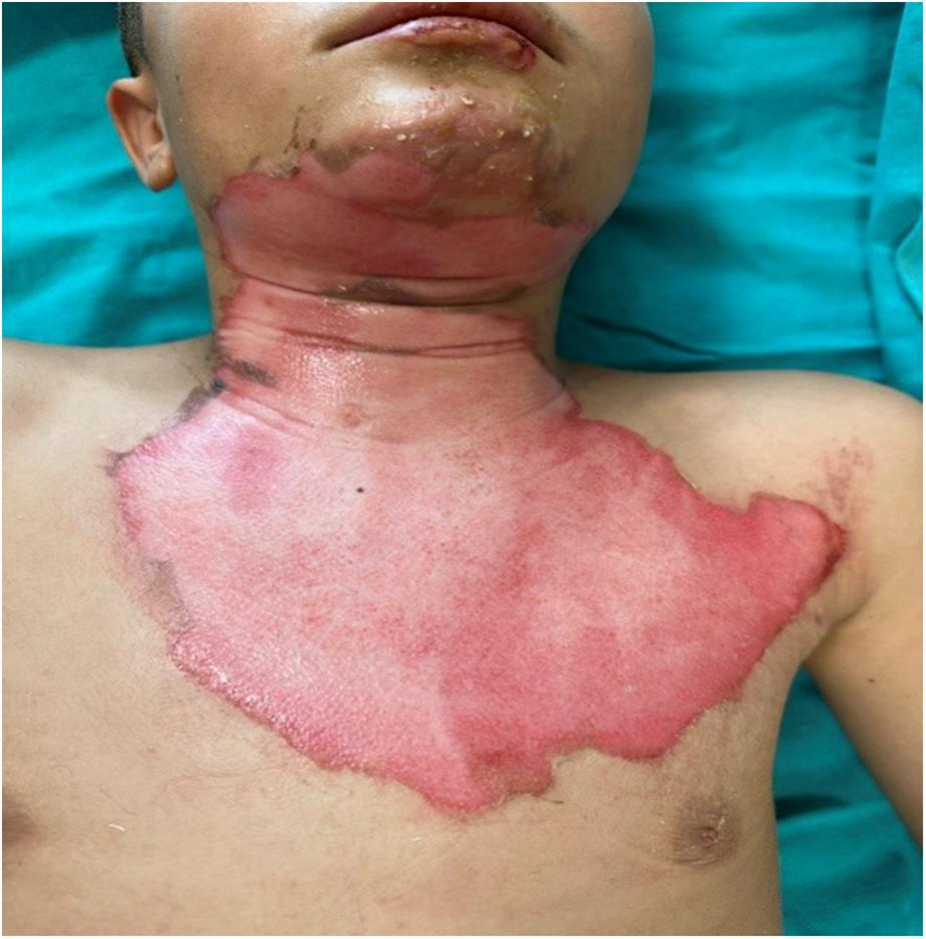
Day 1 clinical image of a pediatric flame burn patient (patient 3, group 2), prior to hemoglobin spray application.

**Figure 2:**
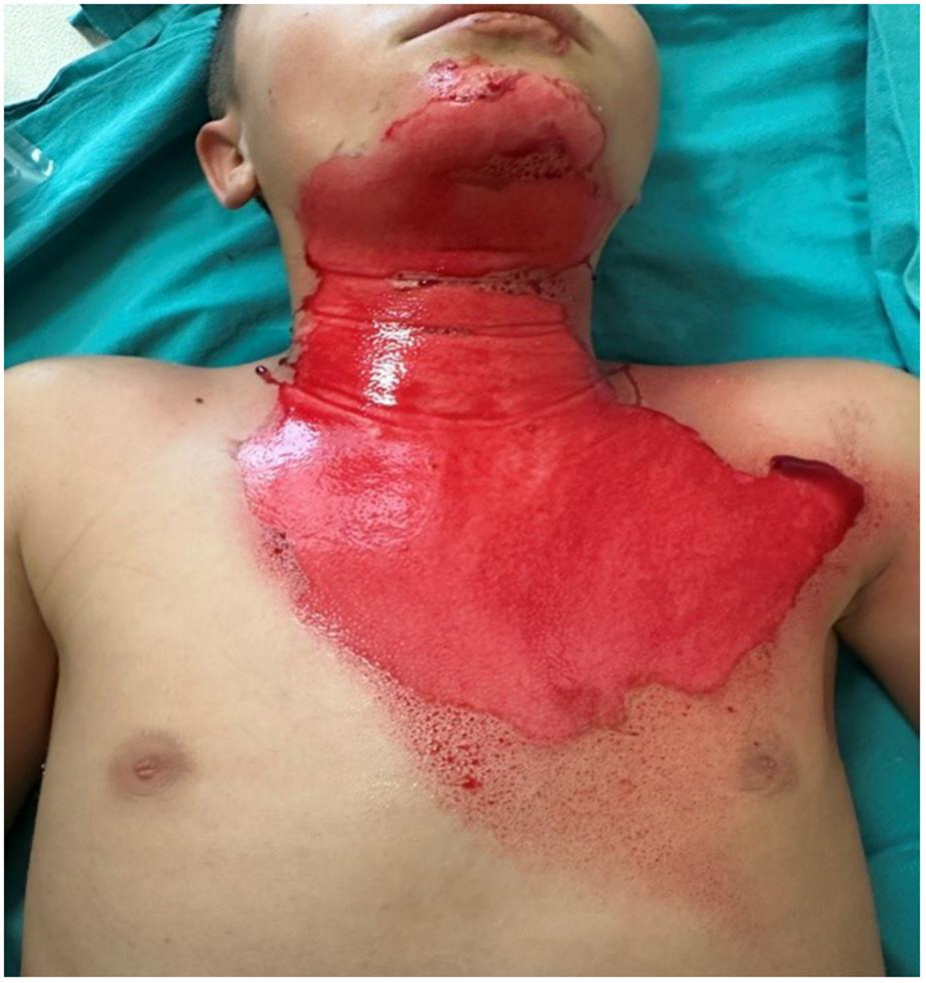
Day 1 clinical image of a pediatric flame burn patient (Patient 3, Group 2), after hemoglobin spray application.

**Figure 3:**
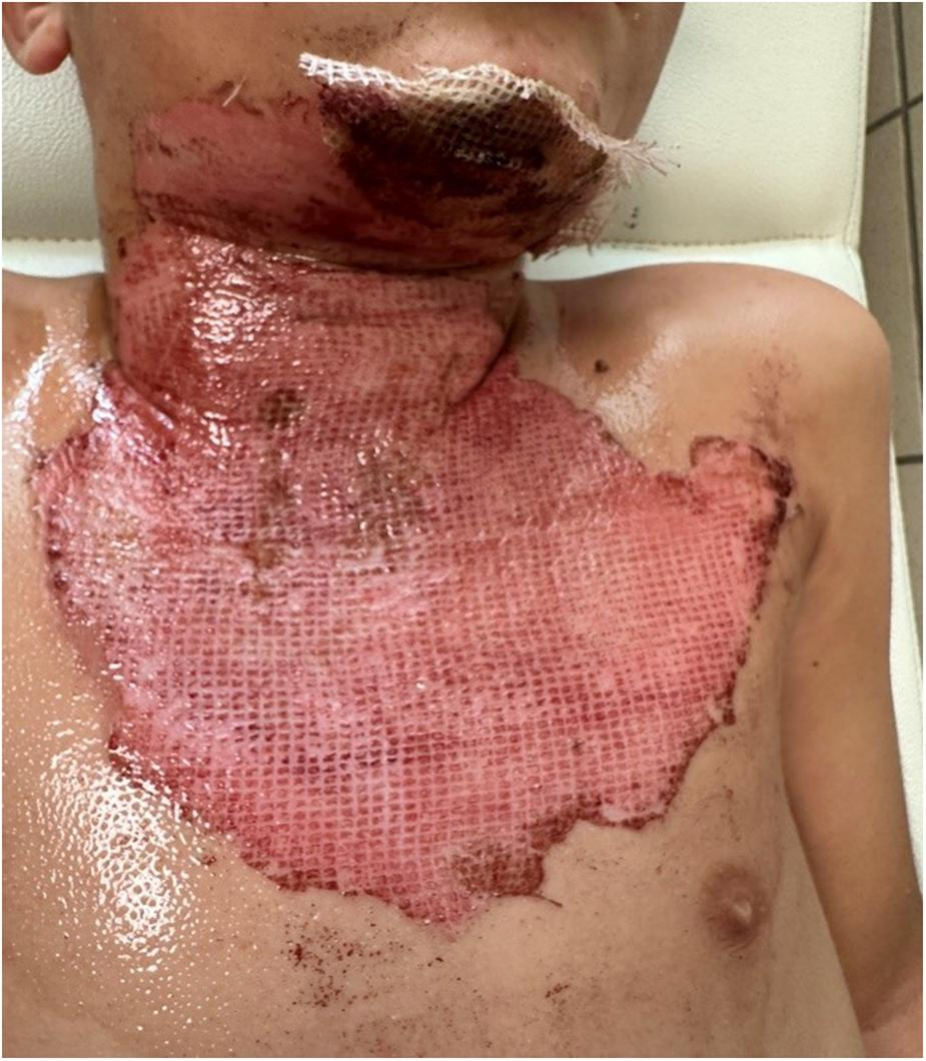
Clinical images on Day 5 post-treatment, showing wound healing progression after hemoglobin spray application in the same patient.

**Figure 4:**
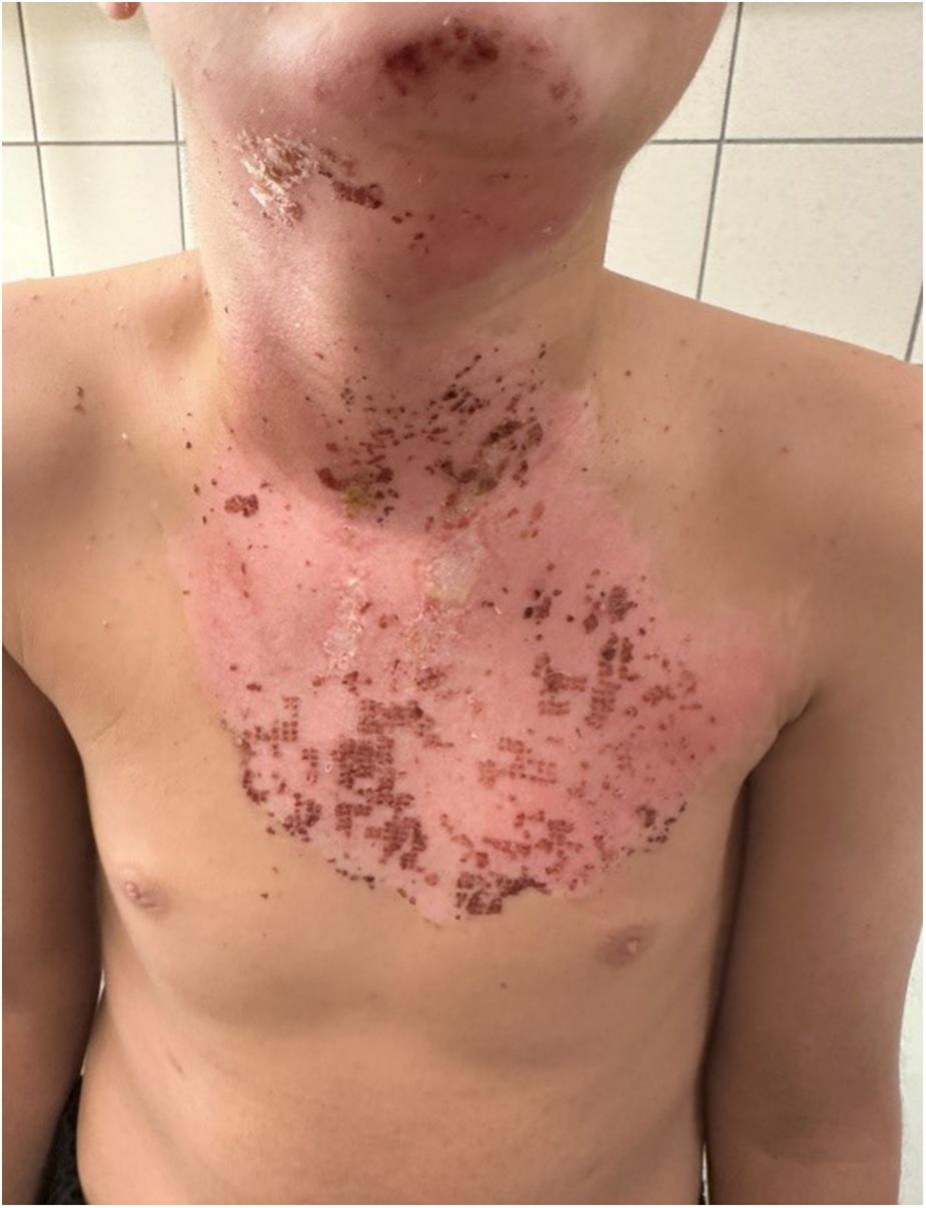
Clinical images on Day 7 post-treatment, showing wound healing progression after hemoglobin spray application in the same patient.

Statistical analysis of the study was completed with 31 patients in Group 1 and 28 patients in Group 2. The demographic and clinical characteristics of the patients are presented in Table 1. The mean percentage of deep second-degree burns was 1.71% ± 1.97% in Group 1, whereas it was 2.71% ± 2.16% in Group 2. A statistically significant difference was identified between the groups with respect to deep second-degree burn percentage according to treatment modality (z = 1.981, *p* = 0.048). No statistically significant differences were observed between the treatment groups in terms of age, sex, burn etiology, total burn surface area (%), burn classification, or second-degree burn values (*p* > 0.05) (Table 1). No bacterial growth was detected in the wound culture samples taken before each dressing in either group. Review of the patient data revealed that none of the patients had malnutrition, inhaled oxygen requirement, or comorbidities. No additional disorders were identified among the patients in either group. All patients included in the study had superficial and deep second-degree burns (Figures 1–4).

**Table 1:** Comparison of demographic and clinical characteristics of patients according to treatment modality.

Variables	Treatment modality			
	Group-1 (*n* = 31)	Group-2 (*n* = 28)	Test statistic	
	Mean ± SD median (Min–Max)	Mean ± SD median (Min–Max)	t; z; *χ*^2^	*p*
Age	5,56 ± 3,58	6,57 ± 4,61	z = 0,685	0,494
	4,0 (2–13)	5,5 (1–16)		
Sex, *n* (%)				
Female	10 (32,3)	13 (46,4)	*χ*^2^ = 1,242	0,265
Male	21 (67,7)	15 (53,6)		
Source, *n* (%)				
Hot water	18 (58,1)	20 (71,4)	*χ*^2^ = 1,307	0,520
Concentrated liquid	7 (22,6)	5 (17,9)		
Flame	6 (19,3)	3 (10,7)		
Burn (%)	9,16 ± 2,61	10,14 ± 3,94	t = 1,115	0,270
	9,0 (4–16)	9,5 (4–18)		
Burn classification, *n* (%)				
<10	18 (58,1)	14 (50,0)	*χ*^2^ = 0,385	0,535
≥10	13 (41,9)	14 (50,0)		
2nd degree	7,45 ± 2,16	7,43 ± 2,62	t = 0,037	0,971
	8,0 (2–11)	8,0 (2–14)		
2nd degree deep	1,71 ± 1,97	2,71 ± 2,16	z = 1,981	**0,048**
	2,0 (0–8)	3,0 (0–7)		

Bold values indicate statistical significance (*p* < 0.05).

A total of 77 patients were treated during the study period; however, 11 patients who did not attend follow-up visits and 7 patients with incomplete data were excluded. Among the 11 excluded patients, all had second-degree burns and total body burn surface area (TBSA) involvement of 10.2%. The mean length of hospital stay in this group was 13.6 days. Of these patients, 7 were treated with 99.9% purified hemoglobin spray in addition to paraffin-based wound dressings. Among these 7 patients, 3 had scald burns and 4 had burns caused by concentrated liquids. The mean discharge time for these patients was 7.8 days.

For the remaining 7 excluded patients, burn characteristics were recorded only as second-degree burns in the hospital database, and no further detailed clinical information was available. Consequently, 59 patients (31 in Group 1 and 28 in Group 2) were included in the final analysis.

### 3.2 Hospitalization duration, epithelialization, and dressing frequency

A statistically significant difference was found between the two groups regarding the day of discharge and the day of complete epithelialization (*p* < 0.001). The mean total number of dressings applied to patients in Group 1 was 5.32 ± 1.99, whereas it was 2.32 ± 0.55 in Group 2. There was a statistically significant difference in the total number of dressings between the two groups according to the treatment method (*p* < 0.001).

In our study, we observed that patients in both groups healed without the need for graft or flap procedures. Debridement under anesthesia and escharectomy were required only in cases with delayed wound healing.

### 3.3 Subgroup analysis according to burn percentage

Among patients with a burn percentage of less than 10%, statistically significant differences were observed between the two treatment groups in terms of number of dressings, discharge day, and complete epithelialization day (*p* < 0.05). Similarly, among patients with a burn percentage of 10% or greater, statistically significant differences were found in the total number of dressings, discharge day, and complete epithelialization day (*p* < 0.05) (Table 2). Patients treated with hemoglobin spray had an approximately 56% lower number of dressing changes compared with those receiving conventional dressings. Patients treated with hemoglobin spray had an approximately 44% shorter time to discharge compared with those receiving conventional dressings. Patients treated with hemoglobin spray had an approximately 44% shorter time to complete epithelialization compared with those receiving conventional dressings (Table 3).

**Table 2:** Comparison of individuals’ parameters before discharge by treatment method with burn percentage classification.

Burn percentage	Post-discharge clinical parameter	Treatment method			
		Group-1	Group-2	Test statistics	
		Mean ± SD Median (Min-Max)	Mean ± SD Median (Min-Max)	z	*p*
Burn percentage <10	Total number of dressings applied	4.94 ± 2.13	2.36 ± 0.63	z = 4.492	**<0.001**
		4.0 (3–12)	2.0 (2–4)		
	Need for additional surgical procedures, *n* (%)				
	No	10 (55.6)	12 (85.7)	–	0.073*
	Yes	8 (44.4)	2 (14.3)		
	Day of discharge	9.11 ± 2.05	5.57 ± 2.90	z = 3.996	**<0.001**
		8.5 (6–13)	5.0 (3–15)		
	Day of complete epithelialization	15.39 ± 3.24	8.50 ± 3.13	z = 4.207	**<0.001**
		14.5 (11–24)	8.0 (6–19)		
Burn percentage ≥10	Total number of dressings applied	5.85 ± 1.72	2.29 ± 0.47	z = 4.453	**<0.001**
		6.0 (3–9)	2.0 (2–3)		
	Need for additional surgical procedures, *n* (%)				
	No	4 (30.8)	12 (85.7)	*χ*^2^ = 8.429	**0.004**
	Yes	9 (69.2)	2 (14.3)		
	Day of **d**ischarge	14.23 ± 5.37	7.00 ± 1.75	z = 3.979	**<0.001**
		13.0 (8–26)	7.0 (5–10)		
	Day of **c**omplete **e**pithelialization	19.69 ± 4.91	10.57 ± 2.10	z = 4.329	**<0.001**
		19.0 (13–29)	11.0 (6–14)		

Bold values indicate statistical significance (*p* < 0.05).

z, Mann Whitney U Test; *χ*^2^, Chi-square Test.

* Fisher Exact test results are given.

**Table 3:** Univariate generalized linear model results for total number of dressing changes, day of discharge, and day of complete epithelialization according to treatment method.

Variables	*β*	Standard error	IRR	*p*
Total number of dressing changes				
Intercept	1,672	0,195	**e^−0.830^ ≈0,44**	**<0,001**
Treatment method (Hemoglobin Spray)	−0,830	0,299		**0,006**
Day of discharge				
Intercept	2,421	0,187	**e^−0.583^ ≈0,56**	**<0,001**
Treatment Method (Hemoglobin Spray)	−0,583	0,277		**0,035**
Day of complete epithelialization				
Intercept	2,845	0,184	**e^−0.589^ ≈0,56**	**<0,001**
Treatment method (Hemoglobin Spray)	−0,589	0,271		**0,030**

Bold values indicate statistical significance (*p* < 0.05).

### 3.4 Post-discharge pruritus

Post-discharge pruritus was observed in 87.1% (*n* = 27) of patients in Group 1 and 14.3% (*n* = 4) of patients in Group 2. There was a statistically significant difference in the presence of post-discharge pruritus between the two treatment methods (*p* < 0.001) (Table 4). Among patients with less than 10% total burn surface area, post-discharge pruritus rates differed significantly between the two groups (*p* < 0.001). Similarly, in patients with 10% or greater burn surface area, a statistically significant difference was also observed in the incidence of pruritus in the healed burn area according to treatment method (*p* < 0.001) (Table 5).

**Table 4:** Comparison of post-discharge clinical parameters by treatment method.

Post-discharge clinical parameter	Treatment method			
	Group-1 (*n* = 31)	Group-2 (*n* = 28)	Test statistics	
	*n* (%)	*n* (%)	*χ* ^2^	*p*
Post-discharge pruritus				
No	4 (12.9)	24 (85.7)	*χ*^2^ = 31.279	**<0.001**
Yes	27 (87.1)	4 (14.3)		
Post-discharge 3rd month pigmentation defect				
None	1 (3.2)	10 (35.7)	*χ*^2^ = 10.238	**0.001**
Present	30 (96.8)	18 (64.3)		
Post-discharge 3rd month epithelialization defect				
None	22 (71.0)	27 (96.4)	–	**0.010** *
Present	9 (29.0)	1 (3.6)		
Was there a pigmentation defect in the 6th month?				
None	5 (16.1)	19 (67.9)	*χ*^2^ = 16.313	**<0.001**
Present	26 (83.9)	9 (32.1)		
Did hypertrophic scar develop during 6-month follow-ups?				
No	20 (64.5)	27 (96.4)	*χ*^2^ = 9.247	**0.002**
Yes	11 (35.5)	1 (3.6)		
Did contracture develop during 6-month follow-ups?				
No	27 (87.1)	28 (100.0)	–	0.069*
Yes	4 (12.9)	0 (0.0)		

*χ*^2^, Chi-square Test.

Bold values indicate statistical significance (*p* < 0.05).

* Fisher Exact test results are given.

**Table 5:** Comparison of individuals’ parameters after discharge by treatment method in case of burn percentage classification.

Burn percentage	Post-discharge clinical parameter	Treatment method			
		Group-1	Group-2	Test statistics	
		*n* (%)	*n* (%)	χ^2^	*p*
Burn percentage <10	Post-discharge pruritus				
	No	3 (16.7)	12 (85.7)	*χ*^2^ = 15.077	**<0.001**
	Yes	15 (83.3)	2 (14.3)		
	Post-discharge 3rd month pigmentation defect				
	None	1 (5.6)	3 (21.4)	–	0.210*
	Present	17 (94.4)	11 (78.6)		
	Post-discharge 3rd month epithelialization defect				
	None	14 (77.8)	14 (100.0)	–	0.085*
	Present	4 (22.2)	0 (0.0)		
	Was there a pigmentation defect in the 6th month?				
	None	4 (22.2)	10 (71.4)	*χ*^2^ = 7.748	**0.005**
	Present	14 (77.8)	4 (28.6)		
	Did hypertrophic scar develop during 6-month follow-ups?				
	No	12 (66.7)	14 (100.0)	–	**0.020** *
	Yes	6 (33.3)	0 (0.0)		
	Did contracture develop during 6-month follow-ups?				
	No	15 (83.3)	14 (100.0)	–	0.165*
	Yes	3 (16.7)	0 (0.0)		
Burn percentage ≥10	Post-discharge pruritus				
	No	1 (7.7)	12 (85.7)	*χ*^2^ = 16.436	**<0.001**
	Yes	12 (92.3)	2 (14.3)		
	Post-discharge 3rd month pigmentation defect				
	None	0 (0.0)	7 (50.0)	–	**0.004** *
	Present	13 (100.0)	7 (50.0)		
	Post-discharge 3rd month epithelialization defect				
	None	8 (61.5)	13 (92.9)	–	0.067*
	Present	5 (38.5)	1 (7.1)		
	Was there a pigmentation defect in the 6th month?				
	None	1 (7.7)	9 (64.3)	–	**0.003** *
	Present	12 (92.3)	5 (35.7)		
	Did hypertrophic scar develop during 6-month follow-ups?				
	No	8 (61.5)	13 (92.9)	–	0.067*
	Yes	5 (38.5)	1 (7.1)		
	Did contracture develop during 6-month follow-ups?				
	No	12 (92.3)	14 (100.0)	–	0.481*
	Yes	1 (7.7)	0 (0.0)		

*χ*, Chi-square Test.

Bold values indicate statistical significance (*p* < 0.05).

* Fisher Exact test results are given.

### 3.5 Pigmentation defects and hypertrophic scar development

**Figure 5:**
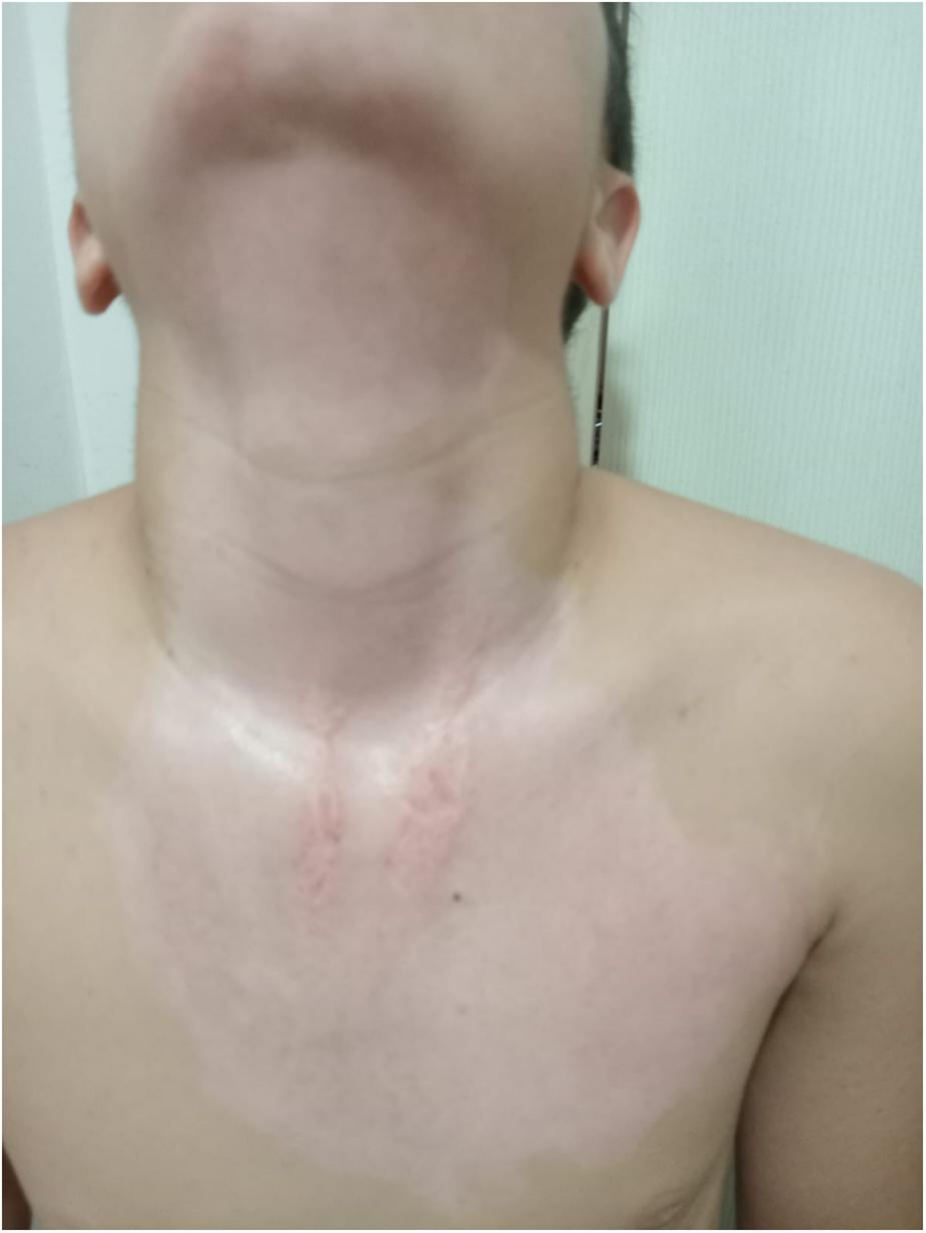
Clinical image on Day 30 of the same patient, demonstrating advanced epithelialization.

**Figure 6:**
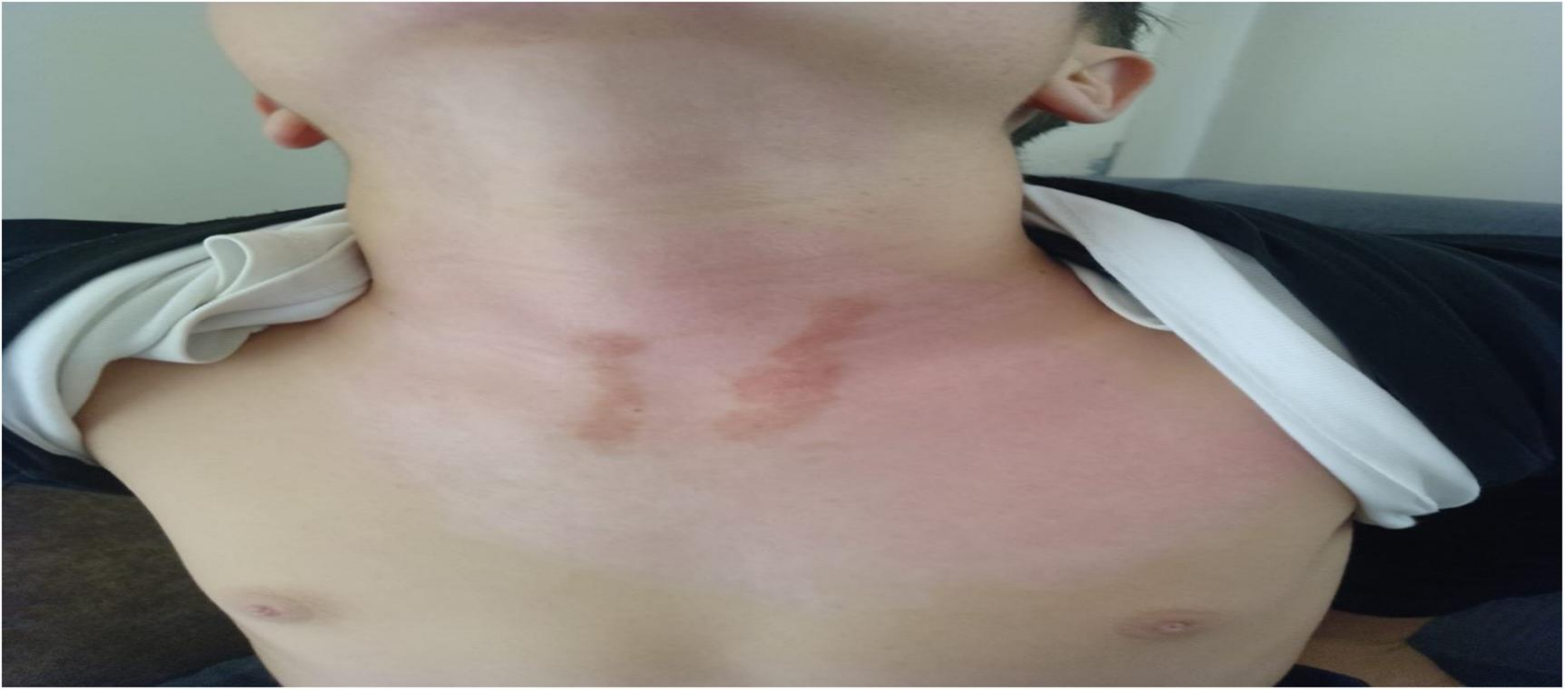
Six-month follow-up image of the same patient, showing the final healing status and scar maturation.

At the third month after discharge, there was a statistically significant difference between the two groups in terms of pigmentation defects and hypertrophic scar development (*p* < 0.05) (Table 4) (Figures 5, 6).

No systemic or local allergic reactions related to the use of purified hemoglobin spray were observed during or after the treatment period.

### 3.6 Univariable logistic regression analysis

According to the univariable logistic regression analyses, the use of hemoglobin spray was associated with a substantially lower risk of several adverse outcomes compared with conventional dressings. Specifically, the risk of requiring an additional surgical intervention was reduced by 86.3% (1–0.137) in patients treated with hemoglobin spray. Similarly, the risk of post-discharge pruritus was reduced by 97.5% (1–0.025). The risk of pigmentation defects at the third month after discharge was reduced by 94.0% (1–0.060), while the risk of epithelialization defects at the third month was reduced by 90.9% (1–0.091). At the sixth month, the risk of pigmentation defects was reduced by 90.9% (1–0.091), and the risk of hypertrophic scar development during the 6-month follow-up period was reduced by 93.3% (1–0.067) in the hemoglobin spray group.

### 3.7 Multivariable and sensitivity analyses

After adjustment for age, percentage of total body surface area burned (%TBSA), and sex, the use of hemoglobin spray remained independently associated with a reduced risk of post-discharge pruritus, with a 96.5% (1–0.040) lower risk compared with conventional dressings. Multivariable Firth penalized logistic regression analyses were performed by including potential confounders such as age, %TBSA, and sex in the model. This method was preferred to account for the small sample size and to reduce the risk of separation. In multivariable analyses, post-discharge pruritus continued to demonstrate a strong and independent association with hemoglobin spray use (Table 6).

**Table 6:** Univariable and multivariable firth penalized logistic regression results for post-treatment outcomes according to treatment method.

Outcomes	Univariable		Multivariable	
	*p*	OR (%95 CI)	*p*	Adj OR (%95 CI)
Need for additional surgical intervention	**0,002**	0,137 (0,038–0,490)	0,669	0,634 (0,07–5,57)
Post-discharge pruritus	**<0,001**	0,025 (0,006–0,110)	**0,002**	0,04 (0,001–0,26)
Pigmentation defect at 3 months after discharge	**0,010**	0,060 (0,007–0,508)	0,215	0,06 (0,001–3,16)
Epithelialization defect at 3 months after discharge	**0,028**	0,091 (0,011–0,770)	0,833	0,73 (0,007–12,10)
Pigmentation defect at 6 months	**<0,001**	0,091 (0,026–0,316)	0,701	0,69 (0,09–5,00)
Development of hypertrophic scarring during the 6-month follow-up	**0,013**	0,067 (0,008–0,565)	0,054	0,04 (0,01–1,05)
Development of contracture during the 6-month follow-up	0,998	0,001 (0,001-)		

OR, odds ratio.

Bold values indicate statistical significance (*p* < 0.05).

Group comparisons were conducted according to the groups to which patients were initially assigned, in accordance with the intention-to-treat principle. To assess the robustness of the findings, sensitivity analyses were performed using alternative statistical methods and model assumptions. Across different analytical approaches, the direction of the observed effects remained unchanged, and the results were consistent with those of the primary analysis (Table 7).

**Table 7:** Intention-to-treat analysis of dressing frequency, discharge day, and epithelialization time according to treatment method.

Outcomes	Treatment method				
	Group-1 (*n* = 31)	Group-2 (*n* = 28)		GLM (Negative Binominal)	GLM (Poisson)
	Mean ± SD Median (Min–Max)	Mean ± SD Median (Min–Max)	p*		
Total number of dressing changes	5.32 ± 1.99	2.32 ± 0.55	**<0** **.** **001**	**IRR** **=** **0,56 *p*** **=** **0,006**	**IRR** **=** **0,56 *p*** **=** **0,006**
	5.0 (3–12)	2.0 (2–4)			
Day of discharge	11.26 ± 4.53	6.29 ± 2.46	**<0**.**001**	**IRR** **=** **0,44 *p*** **=** **0,035**	**IRR** **=** **0,56 *p*** **=** **0,006**
	10.0 (6–26)	5.5 (3–15)			
Day of complete epithelialization	17.19 ± 4.49	9.54 ± 2.82	**<0**.**001**	**IRR** **=** **0,44 *p*** **=** **0,030**	**IRR** **=** **0,56 *p*** **=** **0,006**
	16.0 (11–29)	9.0 (6–19)			

Bold values indicate statistical significance (*p* < 0.05).

*p value for the comparison between Group 1 and Group 2.

## 4 Discussion

In this study, the effects of using 99.9 percent purified hemoglobin spray from the first dressing on burn healing were evaluated in patients with burns treated in the pediatric burn unit. Since purified hemoglobin spray has only recently begun to be applied in pediatric patients during the early phase, the number of patients with long-term results remains limited. The spray is used in superficial second-degree burns. In third- or fourth-degree burns, its effectiveness may be limited due to complete damage to the dermis.

Patients with a burn area of less than 30 percent are hospitalized and treated in the pediatric burn unit. There is a need to evaluate the effectiveness of the treatment in patients with higher burn percentages. It was observed that both the length of hospital stay and the epithelialization time were shorter in Group 2 patients. In the literature, the average length of stay for patients with superficial second-degree burns reported to range between 10 and 17 days (6, 25, 26). Prolonged hospitalization could have cause psychological effects on both patients and parents (27, 28). Therefore, burn care performed with purified hemoglobin spray may improve quality of life and reduce the occurrence of psychiatric disorders in parents.

Although pruritus was known to occur more frequently in patients with large burn areas, it is also common in those with small burns (20, 29). In our study, regardless of burn percentage, pruritus was less frequent in Group 2 patients. This may be due to the prevention of tissue hypoxia in the early period, allowing more regular nerve regeneration. Purified hemoglobin spray increased collagen synthesis by promoting hydroxylation of proline and lysine, contributing to the formation of a stable collagen structure (16). Thus, it may shorten or even prevent the duration of neuropathic pruritus.

Other epithelialization defects observed after burns include pigmentation differences and hypertrophic scarring. Hypertrophic scar formation occured as a result of irregular collagen synthesis and is common in pediatric patients with burns. This condition imposes both a physical and psychological burden. It was known that rapid epithelialization helps prevent hypertrophic scar formation (12). In our study, pigmentation differences and hypertrophic scar rates were significantly lower in Group 2 patients at the 3rd- and 6th-month follow-ups. Early hypertrophic scarring typically develops between 3 and 6 months, whereas late hypertrophic scarring occurs between 12 and 18 months after injury. In our study, we reported the outcomes related to early hypertrophic scar formation during the follow-up period. However, we haven't yet analyzed the data regarding long-term follow-up outcomes. Therefore, our knowledge regarding the effects of purified hemoglobin spray on late hypertrophic scar development remained limited (30). Silicone sheets or gel-based pressure garments were considered first-line prophylactic and therapeutic options for hypertrophic scars and keloids (31). However, several validated scar assessment scales were available to ensure accurate diagnosis and appropriate management. The Seattle Scar Scale was a photographic assessment method that compared scar height, surface characteristics, color, and thickness with the surrounding normal skin and had been validated in the pediatric patients with burns population. The Vancouver Scar Scale (VSS) and the Patient and Observer Scar Assessment Scale (POSAS) were the most commonly used instruments for the physical evaluation of burn scars (32). Although these conditions were carefully monitored and managed in our patients, we would like to emphasize that, beyond the importance of early outcomes such as hypertrophic scar and keloid development, long-term follow-up using objective and validated scar assessment scales is essential to obtain more reliable and consistent data.

Purified hemoglobin spray had been shown to be effective in the treatment of chronic wounds; however, data on its use in the early phase of burn treatment were limited (4, 12, 24, 33). In this respect, our study represents the first conducted in a pediatric patients with burns population. Early application of purified hemoglobin spray was observed to shorten the epithelialization period. The main reason for this may be the increase in local oxygen levels in the damaged tissue. Oxygen activated growth factors and ensured proper collagen synthesis, which in turn might have prevented infection and hypertrophic scar formation. It was considered that purified hemoglobin spray accelerated healing by increasing oxygenation in the damaged tissue, enhancing angiogenesis, and reducing the formation of free oxygen radicals when used in addition to conventional dressings, thereby shortening the hospital stay.

In this study, no visual or quantitative method could be used in the laboratory to demonstrate that 99.9 percent purified hemoglobin spray increased the local oxygen level in the tissue. It was considered that this feature should be supported by further clinical trials and experimental studies.

The number of dressings performed for second-degree burns was generally known to range between three and eight (11), and the number of dressings had been shown to be associated with the length of hospital stay (34, 35). In our study, the average number of dressings was two in Group 2 and five in Group 1. This result suggested that the use of purified hemoglobin spray might accelerate epithelialization, reduce the need for dressing changes, and decrease the requirement for additional surgical procedures.

## 5 Limitations

This study had several limitations. The purified hemoglobin spray was a commercial product from a single manufacturer; although the product name was not disclosed, relevant literature using the same product was reviewed. In addition, cellular-level differences in burn depth and tissue involvement could not be quantitatively assessed within the scope of this study.

The study's retrospective and non-randomized design limited causal inference, and selection bias could not be fully excluded. In addition, only hospitalized pediatric patients with second-degree burns and TBSA <30% were included, which limited the generalizability of the findings to outpatient cases, more extensive burns, or full-thickness injuries.

Outcome assessment relied on clinical evaluation by a single blinded clinician, and the absence of standardized objective tools (e.g., scar scales, quantitative pruritus measures, or imaging-based assessments) might have affected reproducibility. The proposed mechanism of enhanced tissue oxygenation was not directly measured, and follow-up was limited to six months, which might have been insufficient to capture long-term outcomes.

Cost-effectiveness and patient-reported outcomes could not be assessed due to the limitations of retrospective data. Future prospective studies with objective measures and longer follow-up were needed to better define the clinical impact of purified hemoglobin spray.

Due to the retrospective and single-blinded basic randomization, differences in burn depth or clinician decision-making between groups might have influenced the need for additional surgical procedures.

The absence of a prospectively registered study protocol might have limited transparency regarding outcome selection.

The assessment of complete epithelialization was based on clinical judgment and lacked photographic documentation, blinded review, and inter-rater reliability analysis.

## 6 Conclusion

1. Purified hemoglobin spray used in addition to conventional burn dressings was found to enhance tissue oxygenation, accelerate epithelialization, reduce the need for dressing changes, and shorten the length of hospital stay in pediatric patients with burns.
2. In addition, it was associated with a decrease in pruritus complaints during and after treatment and a reduced risk of hypertrophic scar formation.
3. Because of the retrospective and non-randomized study design, a prospective randomized controlled study was needed to confirm the independent effect of hemoglobin spray on wound healing, length of hospital stay, and scar outcomes.

## Data Availability

The original contributions presented in the study are included in the article/Supplementary Material, further inquiries can be directed to the corresponding author.
