# Supplementary material for: The effect of purified hemoglobin spray on the treatment of pediatric patients with second-degree burns
Source: Front Pediatr. 2026 Feb 20;14:1744372. doi: 10.3389/fped.2026.1744372 (PMC12963345; doi:10.3389/fped.2026.1744372)
Supplement: Supplementary file 1 [file Datasheet1.pdf]

STROBE Statement—Checklist of items that should be included in reports of ***case-control studies***

	Item No	Recommendation
**Title and abstract**	1	(*a*) The effect of purified hemoglobin spray on the treatment of pediatric second-degree burns
		(*b*) This study investigates the use of purified hemoglobin spray as an adjunct topical therapy in pediatric second-degree burns, aiming to enhance tissue oxygenation and accelerate epithelialization.
**Introduction**		
Background/rationale	2	We retrospectively investigated the effect of using 99.9% purified hemoglobin spray as an adjunct to standard burn dressings on early burn wound healing in pediatric patients.
Objectives	3	We aimed to share our clinical observations regarding the effect of the spray, which we believe may alleviate hypoxia in burn tissue at an earlier stage, on this process.* (*Elg F, Hunt S. Hemoglobin spray as adjunct therapy in complex wounds: Meta-analysis versus standard care alone in pooled data by wound type across three retrospective cohort controlled evaluations. *SAGE Open Med*. 2018;6:2050312118784313. Published 2018 Jun 27. doi:10.1177/2050312118784313.)
**Methods**		
Study design	4	Comparative outcomes were presented for second-degree burns treated in hospitalized pediatric patients, in whom conventional wound dressings were used based on clinical observation, with or without the adjunctive use of purified hemoglobin spray.
Setting	5	Training and Research Hospital Burn Unit between December 1, 2023, and December 1, 2024, The follow-up period for the patients was defined as six months.
Participants	6	(*a*) Patients younger than 18 years of age who were hospitalized and treated in the pediatric burn unit for second-degree burns, whose data were accessible through the hospital medical record system, and whose follow-up examination data up to 6 months after discharge were available were included in the study.<br>Second-degree burn patients treated by a single clinician during the specified time period were divided into two groups according to the treatment methods, based on data obtained from the hospital patient record system.
		(*b*) Not applicable, as this was not a matched study.
Variables	7	Outcomes, exposures, predictors, and potential confounders were predefined and clinically assessed. The primary outcome was time to epithelialization, while secondary outcomes included pigmentation changes, hypertrophic scar formation, and length of hospital stay. The main exposure was the use of purified hemoglobin spray. No matching or effect modification was performed.
Data sources/ measurement	8*	Data were obtained retrospectively from the hospital electronic medical record system and clinical follow-up notes. Variables including epithelialization time, pigmentation changes, hypertrophic scar formation, and length of hospital stay were assessed through routine clinical evaluation. All assessments were performed using the same clinical criteria in both groups, ensuring comparability of measurements.
Bias	9	Although the study was retrospective and non-randomized, efforts were made to reduce bias by applying consistent treatment protocols and uniform outcome assessments performed by the same clinician.
Study size	10	Study size was determined by the number of eligible patients available during the study period; no prior sample size calculation was performed.
Quantitative variables	11	Quantitative variables were analyzed using appropriate descriptive and comparative statistical methods. Continuous variables were summarized as mean ± standard

		deviation or median (interquartile range), depending on data distribution. Comparisons between groups were performed using appropriate statistical tests. No arbitrary categorization of continuous variables was applied, except where clinically relevant.
Statistical methods	12	(*a*) Statistical analyses were conducted using appropriate tests based on data distribution. Potential confounders were assessed through baseline comparisons between groups; no multivariable adjustment was performed due to the retrospective design.
		(*b*) No predefined subgroup or interaction analyses were performed due to the retrospective design and limited sample size.
		(*c*) Patients with incomplete medical records or missing follow-up data were excluded from the study. Therefore, no imputation methods were applied for missing data.
		(*d*) No matching of cases and controls was performed, as this was not a matched study.
		(*e*) Sensitivity analyses were not conducted, as the retrospective nature of the study and the limited sample size did not allow for meaningful sensitivity testing.
**Results**		
Participants	13*	(a) Of 77 patients assessed for eligibility, 18 were excluded (11 lost to follow-up and 7 due to incomplete data). A total of 59 patients (31 in Group 1 and 28 in Group 2) were included in the final analysis.
		(b) Eighteen patients were excluded: 11 due to loss to follow-up and 7 due to incomplete data.
		**(c)** Patient Flow Diagram

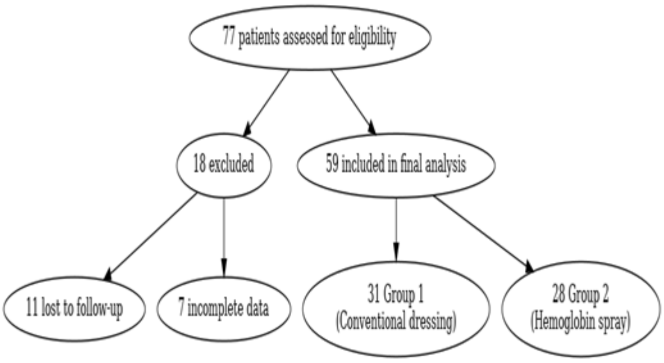

Descriptive data	14*	(a) Participant characteristics and exposure status were summarized in Table 1, including age, sex, TBSA, burn etiology, and burn localization; key potential confounders (TBSA, etiology, localization, depth) were reported.
		(b) There were no missing data for the variables of interest among the patients included in the final analysis. Patients with incomplete clinical or follow-up data were excluded prior to analysis.
Outcome data	15*	Of the 59 patients included in the final analysis, 31 patients were treated with conventional wound dressings (Group 1), and 28 patients received adjunctive treatment with 99.9% purified hemoglobin spray in addition to conventional dressings (Group 2).
Main results	16	(*a*) Unadjusted comparisons between the two groups were performed to evaluate differences in clinical outcomes. Due to the retrospective design and limited sample size, no multivariable adjusted analyses were conducted. Potential confounding factors were assessed descriptively at baseline, and group comparability was evaluated based on these characteristics.
		(*b*) Continuous variables were analyzed as continuous measures and were not categorized. Therefore, no category boundaries were applied.

		(*c*) Adjusted effect estimates were reported using regression analysis; absolute risk translation was not performed.

Other analyses	17	A generalized linear model was used to evaluate the association between treatment modality and clinical outcomes while adjusting for potential confounders. No subgroup or interaction analyses were performed.
**Discussion**		
Key results	18	Adjunctive use of purified hemoglobin spray was associated with improved early wound healing outcomes, including faster epithelialization and reduced early scar formation.
Limitations	19	The retrospective design, limited sample size, and lack of randomization may have introduced selection bias and residual confounding. Tissue oxygenation was not directly measured, and follow-up duration was insufficient to assess long-term outcomes.
Interpretation	20	In line with our objective of evaluating whether adjunctive 99.9% purified hemoglobin spray may improve early wound healing in pediatric second-degree burns, our findings suggest an association between the use of purified hemoglobin spray and improved early clinical outcomes, including faster epithelialization and more favorable short-term scar-related outcomes. However, these results should be interpreted cautiously given the retrospective, non-randomized single-center design, the limited sample size, and the potential for selection bias and residual confounding despite regression modeling. In addition, tissue oxygenation—the proposed biological mechanism—was not directly measured, and therefore mechanistic conclusions remain inferential. Although multiple outcomes were evaluated, raising the possibility of chance findings due to multiplicity, the overall direction of results was generally consistent with the hypothesized benefit of enhanced oxygen availability and with prior evidence supporting the effectiveness of purified hemoglobin spray in wound care, particularly in chronic wounds. Taken together, the present data support the plausibility of a beneficial effect in the early burn phase, but confirmation in larger prospective and ideally randomized studies with objective oxygenation measures and longer follow-up is required to establish causality and long-term impact.
Generalisability	21	The generalisability of the findings may be limited by the single-center design and specific pediatric population; however, the results may be applicable to similar clinical settings.
**Other information**		
Funding	22	This study received no external funding.

Data were reported separately for each study group.
